# Biotechnological Approaches to Study Plant Responses to Stress

**DOI:** 10.1155/2013/654120

**Published:** 2012-12-30

**Authors:** Rosa M. Pérez-Clemente, Vicente Vives, Sara I. Zandalinas, María F. López-Climent, Valeria Muñoz, Aurelio Gómez-Cadenas

**Affiliations:** Department of Agricultural Sciences, Universitat Jaume I, Campus Riu Sec, 12071 Castelló de la Plana, Spain

## Abstract

Multiple biotic and abiotic environmental stress factors affect negatively various aspects of plant growth, development, and crop productivity. Plants, as sessile organisms, have developed, in the course of their evolution, efficient strategies of response to avoid, tolerate, or adapt to different types of stress situations. The diverse stress factors that plants have to face often activate similar cell signaling pathways and cellular responses, such as the production of stress proteins, upregulation of the antioxidant machinery, and accumulation of compatible solutes. Over the last few decades advances in plant physiology, genetics, and molecular biology have greatly improved our understanding of plant responses to abiotic stress conditions. In this paper, recent progresses on systematic analyses of plant responses to stress including genomics, proteomics, metabolomics, and transgenic-based approaches are summarized.

## 1. Introduction

Plants and animals share some response mechanisms to unfavorable environmental conditions; however, plants, being sessile organisms, have developed, in the course of their evolution, highly sophisticated and efficient strategies of response to cope with and adapt to different types of abiotic and biotic stress imposed by the frequently adverse environment.

Stress can be understood as a stimulus or influence that is outside the normal range of homeostatic control in a given organism: if a stress tolerance is exceeded, mechanisms are activated at molecular, biochemical, physiological, and morphological levels; once stress is controlled, a new physiological state is established, and homeostasis is reestablished. When the stress is retired, the plant may return to the original state or to a new physiological situation [1]. 

In the last years, and because of the great interest for both basic and applied research, there has been an important progress in the understanding of the mechanisms and processes underlying abiotic stress adaptation and defense in different plant species [1, 2]. The sensing of biotic or abiotic stress conditions induces signaling cascades that activate ion channels, kinase cascades, production of reactive oxygen species (ROS), accumulation of hormones such as salicylic acid, ethylene, jasmonic acid, and abscisic acid. These signals ultimately induce expression of specific subsets of defense genes that lead to the assembly of the overall defense reaction [3].

**Figure 1:**
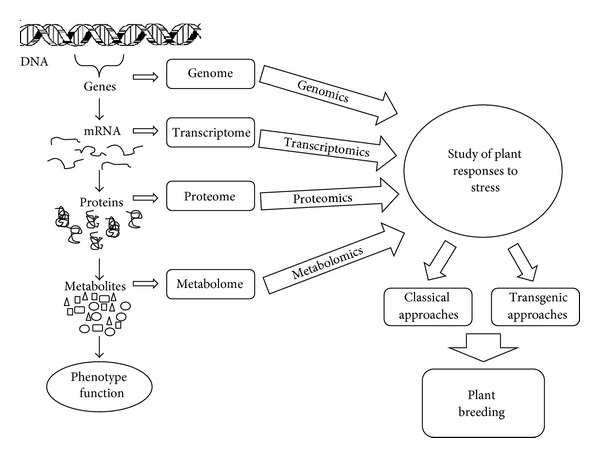
Plant response to abiotic stress factors. Genomics, transcriptomics, proteomics, and metabolomics have enabled active analyses of regulatory networks that control abiotic stress responses. Such analyses increase our knowledge on plant responses and adaptation to stress conditions and allow improving plant breeding.

The emergence of the novel “omics” technologies, such as genomics, proteomics, and metabolomics, is now allowing researchers to identify the genetic behind plant stress responses (Figure 1). These omics technologies enable a direct and unbiased monitoring of the factors affecting plant growth and development and provide the data that can be directly used to investigate the complex interplay between the plant, its metabolism, and also the stress caused by the environment or the biological threats (insects, fungi, or other pathogens). Plant responses to stress are mediated via profound changes in gene expression which result in changes in composition of plant transcriptome, proteome, and metabolome [4]. 

In this work, the main biotechnological approaches to study plant responses to stress are reviewed. 

## 2. Genomics

A gene by gene approach has been typically used to understand its function. In Table 1, some of the genes involved in plant responses to stress are listed. Functional genomics allows large-scale gene function analysis with high throughput technology and incorporates interaction of gene products at cellular and organism level. The information coming from sequencing programs is providing enormous input about genes to be analyzed. The availability of many plant genomes nowadays (reviewed in [5, 6]) facilitates studying the function of genes on a genomewide scale. The lack of information from other plant genomes will also be compensated in part by the availability of large collection of expressed sequence tags (ESTs) and cDNA sequences [7]. The basic interest behind these EST projects is to identify genes responsible for critical functions. ESTs, cDNA libraries, microarray, and serial analysis of gene expression (SAGE) are used to analyze global gene expression profiles in a functional genomics program. Large mutant collections are tools that complement large-scale expression studies. Gene identification through physical and chemical mutagens has become amenable for large-scale analysis with the availability of markers [8], but gene tagging is more promising for functional analysis on a wider scale. Moreover, the understanding of the complexity of stress signaling and plant adaptive processes would require the analysis of the function of numerous genes involved in stress response.

Numerous investigations show that plant defense response genes are transcriptionally activated by pathogens and also by different types of abiotic stress. It has been described that the induction of specific defense genes, in the response against certain pathogens, is dependent on specific environmental conditions, suggesting the existence of a complex signaling network that allows the plant to recognize and protect itself against pathogens and environmental stress [3]. Similar induction patterns of members of the 14.3.3 gene family (GF14b and GF14c) by abiotic and biotic stresses such as salinity, drought, ABA, and fungal inoculation have been documented in rice [9]. The rice GF14 genes contain cis-elements in their promoter regions that are responsive to abiotic stress and pathogen attack. The 14-3-3s family genes are also subject to the regulation by certain transcript factors [9].

On the other hand, kinase cascades of the mitogen-activated protein kinase (MAPK) class play a remarkably important role in plant signaling of a variety of abiotic and biotic stresses, and it is an essential step in the establishment of resistance to pathogens [10]. It has been described that in Arabidopsis MEKK1 and ANP1 act in the environmental stress response [11, 12], and MPK3, MPK4, and MPK6, are activated by a diversity of stimuli including abiotic stresses, pathogens, and oxidative stress [13].

Elucidating the molecular mechanism that mediates the complex stress responses in plants system is an important step to develop improved variety of stress tolerant crops. Many crop traits are quantitative, complex, and controlled by multiple interacting genes. Recent progress in molecular biology provides the tools to study the genetical make-up of plants, which allows us to unravel the inheritance of all traits whether they are controlled by single genes or many genes acting together, known as the quantitative trait loci (QTL). The molecular marker technologies available since the 1980s allows dissecting the variation in traits. With the progress of QTL mapping, new breeding approaches such as marker-assisted selection and breeding by design have emerged [14]. 

Advances in plant genomics research have opened up new perspectives and opportunities for improving crop plants and their productivity. The genomics technologies have been found useful in deciphering the multigenicity of biotic and abiotic plant stress responses through genome sequences, stress-specific cell and tissue transcript collections, protein and metabolite profiles and their dynamic changes, protein interactions, and mutant screens.

## 3. Proteomics

The adaptation of plants to biotic or abiotic stress conditions is mediated through deep changes in gene expression which result in changes in composition of plant transcriptome, proteome, and metabolome. Since proteins are directly involved in plant stress response, proteomics studies can significantly contribute to elucidate the possible relationships between protein abundance and plant stress acclimation. Several studies [15] have already proven that the changes in gene expression at transcript level do not often correspond with the changes at protein level. The investigation of changes in plant proteome is highly important since proteins, unlike transcripts, are direct effectors of plant stress response. Proteins not only include enzymes catalyzing changes in metabolite levels, but also include components of transcription and translation machinery (Table 2).

In the last years, there has been an important progress in the knowledge of several families of plant transcription factors linked to plant stress responses, such as responses to ultraviolet light, wounding, anaerobic stress, and pathogens [16]. The most important ones are as follows.

- The ethylene-responsive-element-binding factors (ERFs). This protein family has been linked to a wide range of stresses; the RNA levels of specific ERF genes are regulated by cold, drought, pathogen infection, wounding or treatment with ethylene, SA or JA [17]. ERF proteins are shown to function as either activators or repressors of transcription, which is of great relevance in all processes related to plant development and its responses to adverse growing conditions due to both biotic and abiotic factors [18]. It has been reported that ERF proteins from one plant species function in other plant species, enhancing their potential utility in increasing the stress tolerance of plants [19, 20]. However, constitutive overexpression of *ERF *genes generally causes deleterious effects. To overcome this problem, the use of stress-inducible promoters to control the expression of the *ERF* genes has been successfully used (reviewed in [21]).
- NAC proteins are plant-specific transcription factors having a variety of important functions not only in plant development but also in abiotic stress tolerance [22]. NAC domain-containing proteins represent one of the largest TF families, firstly identified in model plants as Arabidopsis and rice but also recently characterized in woody fruit species [23].
- Another important family of transcription factors is the called “basic-domain leucine-zipper (bZIP)” which are regulators of important plant processes such as organ and tissue differentiation [24], cell elongation [25], nitrogen/carbon balance control [26], pathogen defense [27], energy metabolism [28], unfolded protein response [29], hormone and sugar signaling [30], light response [31], osmotic control [32], and seed storage protein gene regulation [33]. One class of bZIP proteins that is linked to stress responses comprises the TGA/*octopine synthase* (*ocs*)-element-binding factor (OBF) proteins. These bind to the *activation sequence-1* (*as-1*)/*ocs* element, which regulates the expression of some stress-responsive genes [34]. A major advance was the discovery that TGA/OBF family members interact with nonexpressor of PR1 (NPR1), a key component in the SA defense signaling pathway [35].
- WRKY proteins are a family of transcription factors that are unique to plants specific WRKY family members show enhanced expression and/or DNA-binding activity following induction by a range of pathogens, defense signals, and wounding (reviewed in [36]). Significant progress has been made in the past years in identifying target genes for WRKY factors. WRKY proteins bind to the W box, which is found in the promoters of many plant defense genes [37]. WRKY proteins also regulate the expression of regulatory genes such as receptor protein kinases [38]. Positive and negative regulation of *WRKY* promoters by specific WRKY proteins has been observed, and the promoters of many of the pathogen- and/or SA-regulated *AtWRK* genes are rich in W boxes [39].
- MYB proteins are key factors in regulatory networks controlling development [40], metabolism [41], and responses to biotic and abiotic stresses [42]. Since the Arabidopsis genome sequence was published, some years ago, an important amount of data has accumulated on the roles of MYB transcription factors in plants and some members of this family are involved in these responses. Therefore, *AtMYB30* encodes an activator of the hypersensitive cell death program in response to pathogen attack [43]; *AtMYB96* acts through the ABA signaling cascade to regulate water stress and disease resistance [44]. *AtMYB33* and *AtMYB101 *are involved in ABA-mediated responses to environmental signals. *AtMYB15* is also involved in cold stress tolerance [45]. *AtMYB108* in both biotic and abiotic stress responses [46]. The elucidation of MYB protein function and regulation that is possible in Arabidopsis will allow predicting the contributions of MYB proteins to the responses to biotic and abiotic stress conditions in other plant species.
- MYC proteins are involved in the response of plants to unfavorable environmental conditions. This transcription factor family plays a role in the induction of apoptosis, important in the hypersensitive cell death program in response to pathogen attack. Another putative MYC target is the ornithine decarboxylase gene, involved in polyamines synthesis. On the other hand, MYC proteins activate the major ABA-dependent stress response (reviewed in [47]).

Therefore, studies of plant reaction upon stress conditions at protein level can significantly contribute to our understanding of physiological mechanisms underlying plant stress tolerance. Proteomics studies could thus lead to identification of potential protein markers whose changes in abundance can be associated with quantitative changes in some physiological parameters related to stress tolerance (reviewed in [16]).

## 4. Metabolomics

The possibility of monitoring a complete set of metabolites could largely improve the understanding of many physiological plant processes. This systematic study, defined as “metabolomics,” is intended to provide an integrated view of the functional status of an organism. Besides its use as a breeding or selection tool, metabolomics techniques have also been used to evaluate stress responses in barley [48], Citrus [49], *Medicago truncatula* [50], and *Arabidopsis thaliana* [51]. Some of the metabolites that have been involved in the plant responses to stress are listed in Table 3.

This technological tool, recently developed, includes different approaches, namely, targeted analysis, metabolic fingerprinting, and metabolite profiling.

Targeted analysis is the most developed analytical approach in metabolomics [49]. It is used to measure the concentration of a limited number of known metabolites precisely, by using either gas chromatography (GC) or liquid chromatography (LC) coupled to mass spectrometry (MS) or nuclear magnetic resonance spectroscopy (NMR).

Other approaches using high throughput metabolite analysis focus on a subset of useful information while avoiding the difficulties of comprehensive metabolite characterization; *metabolic fingerprinting *uses signals from hundreds to thousands of metabolites for rapid sample classification via statistical analysis [52]. In the last years, *metabolite profiling *attempts to identify and quantify a specific class or classes of chemically related metabolites that often share chemical properties that facilitate simultaneous analysis (reviewed in [53]).

The metabolome represents the downstream result of gene expression and is closer to phenotype than transcript expression or proteins. Extensive knowledge on metabolic flows could allow assessment of genotypic or phenotypic differences between plant species or among genotypes exhibiting different tolerance to some biotic or abiotic stresses. In addition, target metabolites have been analyzed as nutritional and/or agronomical biomarkers to classify different crop cultivars or to optimize growth conditions [54].

In contrast to high throughput methodology for the analysis of DNA, RNA, and proteins, current strategies for metabolite characterization still face significant obstacles. These challenges are largely caused by the high degree of chemical diversity among metabolite pools as well as the complexity of spatial and temporal distribution within living tissues. Plant metabolomics methodology and instrumentation are being developed at a rapid pace to address these analytical challenges [55].

Like other functional genomics research, metabolomics generates large amounts of data. Handling, processing, and analyzing this data is a clear challenge for researchers and requires specialized mathematical, statistical, and bioinformatic tools [56]. Further developments in this area require improvements in both analytical science and bioinformatics. Development of new analytical techniques is largely focused on increasing resolution and comprehensiveness, increasing speed and throughput of analytical assays and equipment miniaturization.

## 5. Transgenic Approaches: From the Study of Stress Tolerance Mechanisms in Plants to Crop Genetic Improvement

Use of modern molecular biology tools for elucidating the control mechanisms of stress tolerance and for engineering stress tolerant plants is based on the expression of specific stress-related genes. To date, successes in genetic improvement of environmental stress resistance have involved manipulation of a single or a few genes involved in signaling/regulatory pathways or that encode enzymes involved in these pathways [57].

The plant hormone abscisic acid (ABA) regulates the adaptive response of plants to environmental stresses such as drought, salinity, and chilling via diverse physiological and developmental processes [58]. The ABA biosynthetic pathway has been deeply studied, and many of the key enzymes involved in ABA synthesis have been used in transgenic plants to improve abiotic stress tolerance [59]. Transgenic plants overexpressing the genes involved in ABA synthesis showed increased tolerance to drought and salinity stress [59]. Similarly, many studies have illustrated the potential of manipulating *CBF/DREB* genes to confer improved drought tolerance [60].

Another mechanism involved in plant protection to osmotic stress associated to many abiotic stresses such as drought and salinity implies the accumulation of compatible solutes involved in avoiding oxidative damage and chaperoning through direct stabilization of membranes and/or proteins [61]. Many genes involved in the synthesis of these osmoprotectants have been explored for their potential in engineering plant abiotic stress tolerance [61]. The amino acid proline is known to occur widely in higher plants and normally accumulates in large quantities in response to environmental stresses [62]. The osmoprotectant role of proline has been verified in some crops by overexpressing genes involved in proline synthesis [63]. The results of transgenic modifications of biosynthetic and metabolic pathways in most of the previously mentioned cases indicate that higher stress tolerance and the accumulation of compatible solutes may also protect plants against damage by scavenging of reactive oxygen species (ROS) and by their chaperone-like activities in maintaining protein structures and functions [64]. 

Polyamines, being polycationic compounds of low molecular weight, are involved in many cellular processes, such as replication, transcription, translation, membrane stabilization, enzyme activity modulation, plant growth, and development [65]. It has been reported that stress results in an accumulation of free or conjugated polyamines, indicating that polyamine biosynthesis might serve as an integral component of plant response to stress [66, 67]. 

Polyamines metabolic pathways are regulated by a limited number of key enzymes, among them ornithine decarboxylase (ODC) and arginine decarboxylase (ADC). Transgenic plants overexpressing *ADC* gene showed increase in biomass and better performance under salt stress conditions. It has also been described that genetic transformation with genes encoding ADC improved environmental stress tolerance in various plant species [66].

A common factor among most stresses is the active production of reactive oxygen species [2]. ROS are not only toxic to cells but also play an important role as signaling molecules. Under normal growth conditions, there is equilibrium between the production and the scavenging of ROS, but abiotic stress factors may disturb this equilibrium, leading to a sudden increase in intracellular levels of ROS. 

In order to control the level of ROS and protect the cells from oxidative injury, plants have developed a complex antioxidant defense system to scavenge them [68]. These antioxidant systems include various enzymes and nonenzymatic metabolites that may also play a significant role in ROS signaling in plants. A number of transgenic improvements for abiotic stress tolerance have been achieved through detoxification strategy [69]. These include transgenic plants over expressing enzymes involved in oxidative protection, such as glutathione peroxidase, superoxide dismutase, ascorbate peroxidases, and glutathione reductases [70].

LEA proteins, including several groups of high molecular weight, accumulate in response to different environmental stresses. It has been reported that constitutive overexpression of the HVA1, a group 3 LEA protein from barley, conferred tolerance to soil water deficit and salt stress in transgenic rice plants [71]. It has also been reported that plants expressing a wheat LEA group 2 protein (PMA80) gene or the wheat LEA group 1protein (PMA1959) gene resulted in increased tolerance to dehydration and salt stresses [69].

An important strategy for achieving greater tolerance to abiotic stress is to help plants to reestablish homeostasis under stressful environments, restoring both ionic and osmotic homeostasis. This is a major approach to improve salt tolerance in plants through genetic engineering, where the target is to achieve Na^+^ excretion out of the root, or their storage in the vacuole [72].

Transgenic approaches also aim to improve photosynthesis under abiotic stress conditions through changes in the lipid biochemistry of the membranes. Genetically engineered plants overexpressing chloroplast glycerol-3-phosphate acyltransferase gene (involved in phosphatidyl glycerol fatty acid desaturation) showed an increase in the number of unsaturated fatty acids and a corresponding decrease in the chilling sensitivity [73].

The heat shock response is a highly conserved biological response, occurring in all organisms in response to heat or other toxic agent exposures [74]. Genetic engineering for increased thermotolerance by enhancing heat shock protein synthesis in plants has been achieved in a number of plant species. Some authors have reported the positive correlation between the levels of heat shock proteins and stress tolerance (reviewed in [75]).

A special case of study is the heavy metal contamination. In spite of the natural occurrence of heavy metals as rare elements, diverse anthropogenic practices have contributed to spread them in the environment. Plants have developed mechanisms that can protect cells from heavy metal cytotoxicity, as the cytosolic detoxification by binding to the metal-binding molecules as phytochelatins, and metallothioneins which play an important role in heavy metal detoxification and homeostasis of intracellular metal ions in plant tissues. Overexpression of phytochelatin synthase in Arabidopsis leads to enhanced arsenic tolerance but surprisingly to cadmium hypersensitivity [76]. Therefore, new approaches could contribute to uncovering the complexity of plant tolerance to heavy metal stress [77].

The transcription factors activate cascades of genes that act together in enhancing tolerance towards multiple stresses as indicated before. On the other hand, some stress responsive genes may share the same transcription factors, as indicated by the significant overlap of the gene expression profiles that are induced in response to different stresses [37]. Transcriptional activation of stress-induced genes has been possible in transgenic plants over expressing one or more transcription factors that recognize promoter regulatory elements of these genes [75, 78]. Two families, bZIP and MYB, are involved in ABA signaling and its gene activation. Introduction of transcription factors in the ABA signaling pathway can also be a mechanism of genetic improvement of plant stress tolerance. Constitutive expression of *ABF3 *or *ABF4* demonstrated enhanced drought tolerance in Arabidopsis, with altered expression of ABA/stress-responsive genes, for example, *rd29B*, *rab18*, *ABI1*, and *ABI2* [79].

It is important to point that genetic modification of higher plants by introducing DNA into their cells is a highly complex process. Practically any plant transformation experiment relies at some point on cell and tissue culture. Although the development transformation methods that avoid plant tissue culture have been described for Arabidopsis and have been extended to a few crops, the ability to regenerate plants from isolated cells or tissues *in vitro* is needed for most plant transformation systems. Not all plant tissue is suited to every plant transformation method, and not all plant species can be regenerated by every method [80]. There is, therefore, a need to find both a suitable plant tissue culture/regeneration regime and a compatible plant transformation methodology [81].

## 6. Conclusions

To understand how plants respond to stress, it must be considered that they are subjected to a combination of adverse conditions. This preliminary consideration is essential to understand the performance of plants under stress and also to identify strategies to improve stress tolerance. 

The integration of the omics approaches is likely to enable researchers to reconstruct the whole cascade of cellular events leading to rapid responses and adaptation to the various abiotic stimuli. A well-focused approach combining molecular, physiological, and metabolic aspects of plant stress tolerance is required to increase knowledge on the effects of gene expression and to understand whole plant phenotype under stress. A better understanding of the underlying physiological processes in response to different abiotic stresses can drive the selection of the appropriate promoter or transcription factor to be used for transformation. 

In addition, the use of genetic and genomic analysis to identify DNA molecular markers associated to stress resistance can facilitate breeding strategies for crop improvement. This approach is particularly useful when target characters are controlled by several genes, as in the case of abiotic stress tolerance. These omics approaches could be combined with the potential to map different QTLs contributing to a given agronomical trait and to identify linked molecular markers. This will open the possibility to transfer simultaneously several QTLs and to pyramid QTLs for several agronomical traits in one improved cultivar.

## Figures and Tables

**Table 1:** Genes involved in plant responses to stress.

	Stress	Reference
14.3.3 gene family (GF14b, GF14c)	Salinity, drought, fungal	[9]
MAPK	Abiotic and biotic stresses	[10]
MEKK1 and ANP1	Environmental stress	[11, 12]
MPK3, MPK4 and MPK6	Abiotic stress (pathogens) and oxidative stress	[10, 13]
CBF/DREB families (CBF1, CBF2, DREB2A)	Drought, cold, salinity	[47, 82]
HVA1	Salinity and drought	[83]
Glycerol-3-phosphate acyltransferase gene	Cold	[84]
ICS	Pathogens, UV light	[85]
LOX	Wounding, drought, and pathogens	[86, 87]
bZIPs family (e.g., ABF1, ABF2)	Drought, temperature, salinity	[24–33]
WRKY family (AtWRKY2, AtWRKY6, AtWRKY18)	Pathogens, wounding, salinity, temperature, drought, oxidative stress	[37]
ATAF	Wounding, drought, salinity, cold, pathogens	[88]

**Table 2:** Proteins and enzymes involved in plant responses to stress.

	Stress	Reference
ERF family	Cold, drought, pathogen infection, wounding, ET, SA, and JA	[19]
bZIPs family (e.g., ABF1, ABF2)	Drought, temperature, salt	[24–33]
WRKY	Pathogens, wounding, salinity, temperature, drought, oxidative stress	[36–39]
MYB family (AtMYB15, AtMYB30, AtMYB33 AtMYB60, AtMYB96, AtMYB101 AtMYB15, and AtMYB108)	Biotic and abiotic stress (pathogens, drought, cold)	[40–46]
ABF	Drought	[79]
NAC	Drought, salinity, cold	[22, 23]
MYC	Environmental stresses	[47]
LEA family (PMA 80, PMA 1959)	Salinity and drought	[71]
Heat shock proteins	Temperatures	[74, 75]
LOX family (e.g., LOX1)	Wounding, drought, and pathogens	[86, 87]
Glutathione peroxidase, superoxide dismutase, ascorbate peroxidases, and glutathione reductases	Oxidative stress	[68, 70]

**Table 3:** Metabolites and hormones involved in plant responses to stress.

	Stress	Reference
Abscisic acid, jasmonic acid, salicylic acid, polyamines, and others	Drought, salinity, cold	[3, 58, 65–67]
Proline, glycine-betaine, and other compatible osmolytes	Environmental stresses: drought, salinity, osmotic	[62–64]
Phytoalexins	Microbial pathogens	[89]
Terpenes	Toxins and pathogens	[89]
Phenolic compounds (coumarin, lignin, flavonoids, tannins, isoflavonoids)	Pathogens, oxidative stress, UV light	[89, 90]
Alkaloids	Pathogens (predators)	[87]
Unsaturated fatty acids	Environmental stresses	[73]
ROS, malondialdehyde	Biotic and abiotic stresses	[3, 58, 68]
Phytochelatins and metallothioneins	Heavy metal intoxication	[76, 77]
